# The complete plastome sequence of the endangered orchid *Oberonia japonica* (Orchidaceae)

**DOI:** 10.1080/23802359.2017.1390409

**Published:** 2017-10-17

**Authors:** Young-Kee Kim, Myoung Hai Kwak, Myong Gi Chung, Hoe-Won Kim, Sangjin Jo, Jung-Yeon Sohn, Se-Hwan Cheon, Ki-Joong Kim

**Affiliations:** a Division of Life Sciences, Korea University, Seoul, Korea;; b Department of Plant Resources, National Institute of Biological Resources, Incheon, Korea;; c Division of Life Science and the Research Institute of Natural Science, Gyeongsang National University, Jinju, Korea

**Keywords:** Platome, *Oberonia japonica*, Orchidaceae, endangered species, *ndh* gene family loss

## Abstract

In this study, we report the complete chloroplast sequence of *Oberonia japonica* (Maxim.) Makino (Orchidaceae) (NCBI acc. no. KX871235), which is an endangered plant species currently protected by the National Law of Korea. The gene order and content of the *O. japonica* plastome are similar to those of a typical orchid plastome. The 11 *ndh* genes are pseudogenized or lost completely from the plastome of *O. japonica.* The plastome contains 102 genes, of which 68 are protein-coding genes, 30 and four are transfer RNA (tRNA) and ribosomal RNA (rRNA) genes, respectively. Sixteen genes contain one intron and two genes (*clp*P and *ycf*3) have two introns. The complete plastome is 142,996 bp long and consists of one large and small single copy each of 81,669 and 10,969 bp, respectively, separated by two inverted repeats of 25,179 bp. The AT content of the *O. japonica* plastome is 62.6%. Sixty-five simple sequence repeat (SSR) loci, consisting of 56 mono-SSR, seven di-SSR, and two tri-SSR are scattered along the *O. japonica* plastome. Some of these plastome SSR loci may be useful for developing genetic markers for the *O. japonica* populations. Phylogenetic analysis has shown that *O. japonica* is a member of the Malaxideae. The genus *Oberonia* forms a monophyletic clade with *Dendrobium*.

*Oberonia japonica* (Maxim.) Makino, an epiphytic orchid belonging to the genus *Oberonia*, is native to Korea, Japan, and Taiwan (Lee 2011). It is a small sympodial orchid with elongated spike-like inflorescence. *O. japonica* thrives on the bark of evergreen trees in subtropical forests in Jeju Island of Korea. Less than 100 individual plants in three populations have been reported on the island. The Jeju island population of *O. japonica* corresponds to the northern limit of the distribution range of the species. Therefore, the species was designated as an endangered species, which is protected by the National Law of Korea. The genus *Oberonia* is comprised of approximately 323 species (Chase et al. 2015). *Oberonia* belongs to the subfamily Epidendroideae of the family Orchidaceae in the order Asparagales (APG IV 2016). To develop genetic markers for *O. japonica* for conservation studies, we sequenced and analyzed the plastome of this species. The plastome data will be useful for evaluating the genetic diversity and phylogenetic position of *O. japonica* and related taxa.

The seeds of *O. japonica* were originally collected from a natural population in Jeju Island in Korea. The leaf material of *O. japonica* was collected from a single individual cultivated in laboratory flask from seeds. A voucher specimen and DNA sample were deposited in the Korea University Herbarium (KUS 2015-1269)) and the Plant DNA Bank in Korea (PDBK 2015-1269), respectively. The fresh leaves were ground into a powder in liquid nitrogen, and total DNA was extracted using G-spin™II for plant genomic DNA extraction kit (iNtRON, Seongnam, Korea). The complete plastome was generated using the Illumina MiSeq (San Diego, CA), and assembled using the Geneious 6.1.8 (Kearse et al. 2012). An average coverage of a sequence was 1,011 times the plastome size and, therefore, the sequences were extremely accurate. Annotations were performed using the National Center for Biotechnology Information (NCBI) Basic Local Alignment Search Tool (BLAST) and tRNAscan-SE programs (Lowe and Eddy 1997). The complete plastome sequence was submitted to the NCBI database with accession number KX871235.

The gene order and gene content of *O. japonica* are similar to those of a typical angiosperm (Shinozaki et al. 1986; Kim and Lee 2004; Yi and Kim 2012) except for the 11 *ndh* genes. The 11 *ndh* genes are pseudogenized or lost completely from the plastome of *O. japonica.* The *ndh* gene losses are quite common in the plastomes of Orchidaceae (Chang et al. 2006; Wu et al. 2010; Lin et al. 2015). The plastome of *O. japonica* contains 102 unique genes including 68 protein-coding, 30 transfer RNA (tRNA), and four ribosomal RNA (rRNA) genes. Fourteen genes have a single intron while the *clp*P and *ycf*3 genes have two introns. The length of the complete plastome of *O. japonica* is 142,996 bp, composed of a large single copy (LSC) of 81,669 bp, a small single copy (SSC) of 10,969 of bp, and two inverted repeats (IR) of 25,179 bp. The length of the *O. japonica* plastome is approximately 10 kb shorter than that of the typical angiosperm plastome because of the losses of its *ndh* genes. The average AT content of the plastome is 62.6%. We identified 65 simple sequence repeat (SSR) loci, which consisted of 56 mono-SSR, seven di-SSR, and two tri-SSR scattered along the plastome. Some of these plastome SSR loci may be useful for developing genetic markers for *O. japonica* populations.

We constructed a maximum likelihood (ML) tree to validate the phylogenetic relationships of *O. japonica* among the Orchidaceae. The 82 gene sequences were aligned using MUSCLE in Geneious 6.1.8. The aligned data matrix consists of 70,011 bp. An alignment was used for the phylogenetic analysis using RAxML v. 7.7.1 (Stamatakis et al. 2008). An ML tree was obtained with an ML optimization likelihood value of −310176.363354. *O. japonica* forms a monophyletic group with two species of *Dendrobium.* The *Oberonia-Dendrobium* clade was supported by a 99% bootstrap value (Figure 1). These three species belong to the same Malaxideae tribe within Epidendroideae. The phylogenetic tree obtained in this study using the whole plastome data is similar to that obtained in previous studies, which were based on a few gene sequence data (Cameron 2005).

**Figure 1.**
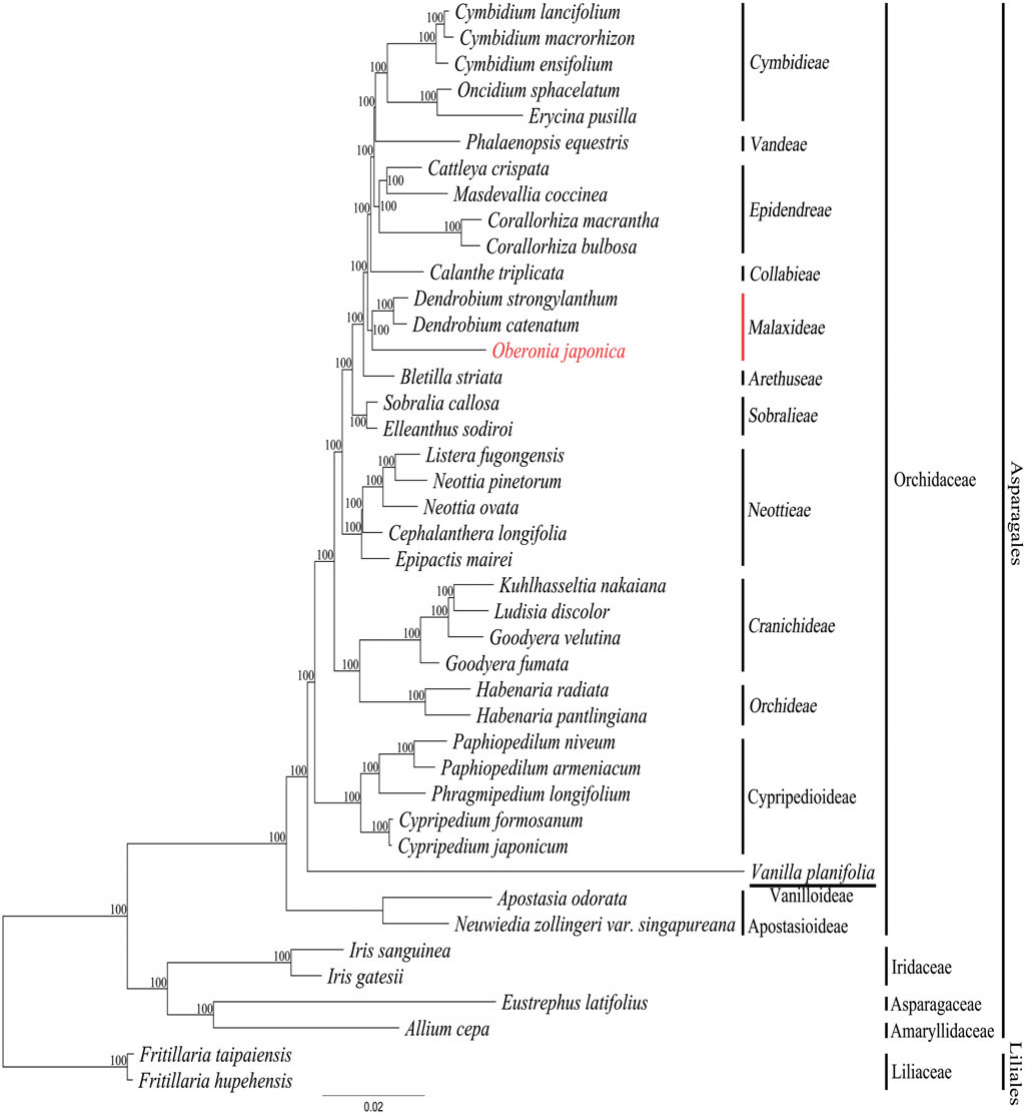
A maximum likelihood (ML) tree of Orchidaceae indicating the phylogenetic position of *Oberonia japonica*. The ML tree is reconstructed from the sequences of 78 protein coding genes and four rRNA genes for 42 plastome sequences. The number above or below of each node indicate bootstrap supporting value from 100 replications. Genbank accession numbers of taxa are shown below; *Allium cepa* (NC024813), *Apostasia odorata* (NC030722), *Bletilla striata* (NC028422), *Calanthe triplicata* (NC024544), *Cattleya crispata* (NC026568), *Cephalanthera longifolia* (NC030704), *Corallorhiza bulbosa* (NC025659), *Corallorhiza macrantha* (NC025660), *Cymbidium ensifolium* (NC028525), *Cymbidium lancifolium* (NC029712), *Cymbidium macrorhizon* (KY354040), *Cypripedium formosanum* (NC026772), *Cypripedium japonicum* (NC027227), *Dendrobium catenatum* (NC024019), *Dendrobium strongylanthum* (NC027691), *Elleanthus sodiroi* (NC027266), *Epipactis mairei* (NC030705), *Erycina pusilla* (NC018114), *Eustrephus latifolius* (NC025305), *Fritillaria hupehensis* (NC024736), *Fritillaria taipaiensis* (NC023247), *Goodyera fumata* (NC026773), *Goodyera velutina* (NC029365), *Habenaria pantlingiana* (NC026775), *Habenaria radiata* (KX871237), *Iris gatesii* (NC024936), *Iris sanguinea* (NC029227), *Kuhlhasseltia nakaiana* (KY354041), *Listera fugongensis* (NC030711), *Ludisia discolor* (NC030540), *Masdevallia coccinea* (NC026541), *Neottia ovata* (NC030712), *Neottia pinetorum* (NC030710), *Neuwiedia zollingeri var. singapureana* (KM244735), *Oberonia japonica* (KX871235), *Oncidium sphacelatum* (NC028148), *Paphiopedilum armeniacum* (NC026779), *Paphiopedilum niveum* (NC026776), *Phalaenopsis equestris* (NC017609), *Phragmipedium longifolium* (NC028149), *Sobralia callosa* (NC028147), *Vanilla planifolia* (NC026778).
